# Serious adverse events associated with bowel preparation for colonoscopy in Japan: Systematic review

**DOI:** 10.1111/den.15055

**Published:** 2025-06-05

**Authors:** Toshihiro Tadano, Koichiro Abe, Seiju Sasaki, Teruhiko Terasawa, Satoyo Hosono, Takafumi Katayama, Keika Hoshi, Tomio Nakayama, Chisato Hamashima

**Affiliations:** ^1^ Cancer Detection Center Miyagi Cancer Society Miyagi Japan; ^2^ Department of Medicine Teikyo University School of Medicine Tokyo Japan; ^3^ Department of Nursing, Faculty of Medical Technology Teikyo University Tokyo Japan; ^4^ Center for Preventive Medicine St. Luke's International Hospital Affiliated Clinic Tokyo Japan; ^5^ Division of Cancer Screening Assessment and Management National Cancer Center Institute for Cancer Control Tokyo Japan; ^6^ Section of General Internal Medicine, Department of Emergency and General Internal Medicine Fujita Health University School of Medicine Aichi Japan; ^7^ Department of Statistics and Computer Science, College of Nursing Art and Science University of Hyogo Hyogo Japan; ^8^ Center for Health Informatics Policy National Institute of Public Health Saitama Japan

**Keywords:** adverse drug event, bowel preparation solution, colonoscopy, laxative, systematic review

## Abstract

**Objectives:**

Bowel preparation for colonoscopy can lead to serious adverse events (AEs), raising significant safety concerns in colorectal cancer (CRC) screening. A systematic review of these serious AEs in Japan was performed to explore potential management strategies.

**Methods:**

The Ovid‐MEDLINE and Ichushi databases were searched from inception to March 2024. Domestic studies that reported serious AEs in adults aged 18 years and older who were administered bowel cleansing agents or laxatives for a scheduled colonoscopy, regardless of its purpose, were extracted. Serious AEs were defined as those requiring hospitalization or extended hospital stays. Selected studies were assessed for quality verification using the established checklist.

**Results:**

A total of 5049 articles were identified through database searches, and 54 articles were extracted based on selection criteria. Reports of the frequency of serious AEs were based on one case series study, which found 13.9 cases of bowel obstruction and 2.3 cases of bowel perforation per 100,000 colonoscopies. Multiple serious AEs caused by different agents were identified in 78 cases across 54 articles. These AEs were predominantly observed in elderly individuals and those with comorbidities. Though most cases were associated with diagnostic tests for symptomatic patients, some were also observed in primary screening or fecal test‐positive individuals. The most common AE was induced by bowel obstruction, primarily in abdominally symptomatic patients, including one fatality.

**Conclusion:**

The frequency and characteristics of serious AEs associated with bowel preparation for colonoscopy in Japan were presented. These findings may contribute to managing these AEs, specifically in CRC screening.

## INTRODUCTION

Colonoscopy is routinely performed for colorectal cancer (CRC) screening, fecal immunochemical test (FIT)‐positive follow‐up, diagnosis of symptoms, surveillance, and polypectomy.^1^, ^2^ Its success depends on adequate bowel preparation.^2^, ^3^, ^4^ In CRC screening, considering that the screening population is asymptomatic and many colonoscopies are performed, ensuring safety from adverse events (AEs) associated with bowel preparation is particularly important.^5^, ^6^ Although serious AEs related to bowel preparation are not common, they can be life‐threatening, and their potential risks should not be ignored.^7^

These AEs are often identified and reported in case series and case reports.^5^ AEs due to bowel preparation for colonoscopy have been reported in various randomized, controlled trials (RCTs) that evaluated the efficacy and safety of bowel cleansing agents and laxatives in asymptomatic populations.^8^, ^9^, ^10^, ^11^ However, these studies predominantly reported only minor AEs, such as nausea and abdominal pain, that did not require admission. Although it is difficult to assess the frequency of these AEs, a British systematic review has documented severe AEs related to bowel preparation for colonoscopy, offering significant insights.^6^ Although severe complications during colonoscopy are typically limited to bleeding, perforation, or cardiac events, severe AEs associated with bowel preparation can vary widely, depending on factors, such as the patient's comorbidities, type of bowel cleansing agents and laxatives, and administration method.^2^, ^3^, ^4^, ^5^, ^6^, ^7^

In Japan, polyethylene glycol electrolyte lavage solution (PEG) has been used for bowel preparation since the 1980s, and other agents have been introduced.^12^, ^13^ As these agents became more common, colonoscopies increased to ~3.0 million annually in clinical settings.^14^ Western guidelines recommended a large‐volume dose of 3–4 L of PEG, but in recent years, split dosing and lower doses have also been used to improve patient tolerance.^2^, ^3^, ^4^ In contrast, Japanese studies confirmed the effect and tolerability of low‐dose regimens, which became standard early on, often combined with laxatives such as sennoside and sodium picosulfate.^13^, ^15^, ^16^, ^17^, ^18^ Despite this safety‐conscious regimen, several Japanese organizations have reported serious AEs related to bowel preparation and issued warnings regarding these AEs.^19^, ^20^, ^21^ Yet the measures taken remain insufficient. Given this background, a systematic review of serious AEs related to bowel preparation for colonoscopy in Japan was conducted to manage them appropriately.

## METHODS

This systematic review was performed as part of an updated evidence review to revise the Japanese guidelines for CRC screening.^22^ Although a study protocol for this review was not registered, it adhered to the PRISMA 2020 statement.^23^ No ethical review was required for a systematic review of published articles.

### Inclusion and exclusion criteria

Studies reporting serious AEs related to the intake of bowel cleansing agents or laxatives in individuals aged 18 years or older scheduled for colonoscopy were considered eligible regardless of its purpose. Serious AEs were defined as those requiring hospitalization or extended hospitalization due to AEs caused by bowel cleansing agents or laxatives, including fatal cases. Given the anticipated paucity of relevant studies, interventional and observational studies, including case reports, were evaluated.

This review was limited to studies conducted in Japan due to potential differences in the type, method, and dosage of bowel‐cleansing agents and laxatives used in other countries. AEs related to bowel preparation before emergency colonoscopy for conditions such as gastrointestinal bleeding were excluded. It is important to note that the absence of reported serious AEs is not equal to a zero‐event frequency in interventional studies with small sample sizes. This potential issue, highlighted in a previous study,^24^ necessitates careful analysis. Therefore, only cases in which serious AEs were reported were evaluated.

### Literature search and selection, data collection

Literature searches were conducted using the Ovid‐MEDLINE and Ichushi databases. The searches were limited to publications in English or Japanese. The search period covered the period from the inception of the databases to April 2024. Search terms included keywords related to “colonoscopy,” “bowel cleansing,” and “AEs.” In the Ichushi database, specific names of commonly used bowel cleansing agents in Japan were also included in the search terms to enhance relevance. Details of the search strategy are provided in Appendix S1. Abstracts of the literature retrieved were screened by pairs of reviewers or by individual reviewers (T.Ta., K.A., and S.S.) to identify potential studies. Discrepancies were resolved by consensus. Full‐text reviews were similarly conducted to narrow eligible studies.

In data collection, the frequency data for AEs were recorded. In addition, for each AE case, patient and clinical characteristics (age, sex, comorbidity, purpose of colonoscopy, types of bowel‐cleansing agents, timing of bowel‐cleansing agent administration, the use of laxatives, types of AEs, main treatment, severity of AEs) were extracted. Obstructive colitis is generally defined as ulcerative inflammatory lesions of the colon caused by obstructive or potentially obstructive lesions.^25^ Therefore, this study recognized it as a complication of bowel obstruction when determining the types of AEs. The severity of AEs was classified according to the National Cancer Institute Common Terminology Criteria for Adverse Events (CTCAE) version 5.^26^ The definitions in this guideline are as follows: Grade 3 refers to serious or medically significant events that are not immediately life‐threatening, for which hospitalization or prolongation of hospitalization is indicated; Grade 4 includes life‐threatening consequences that require urgent intervention; and Grade 5 represents death related to the AE. Each AE was ultimately classified according to the corresponding term based on these principles. In cases of bowel obstruction, the causes, sites, and associated complications were also recorded. Data extraction was performed and cross‐checked (T.Ta. and S.S.).

### Quality assessment and data analysis

The Joanna Briggs Institute (JBI) Critical Appraisal Checklist for Case Series and Case Reports was used to assess the methodological quality and risk of bias of each study to ensure the validity and reliability of the findings included in the analysis.^27^, ^28^ The checklist for case series consists of 10 items, and the checklist for case reports consists of 8 items. The quality of each checklist item was evaluated as “Yes,” “No,” “Unclear,” or “Not applicable,” and the results of the critical appraisal for all questions are presented in tabular form. Two independent investigators (T.Ta. and S.S.) conducted the evaluations. Any discrepancies in the quality assessment were resolved by mutual agreement between the investigators.

AEs induced by colonoscopy were summarized, and clinical characteristics were described. Median and interquartile range values were used for numerical variables, and proportions were calculated for categorical variables. AEs were also analyzed in subgroups divided by age (70 years or over, and under 70 years), the purpose of the colonoscopy, and each type of AE. Fisher's exact test was used to compare patient and clinical characteristics between age groups among patients who experienced serious AEs. *P*‐values are presented for descriptive purposes only, reflecting the observational trend of the data included in this review.

## RESULTS

### Literature search and selection results

**Figure 1:**
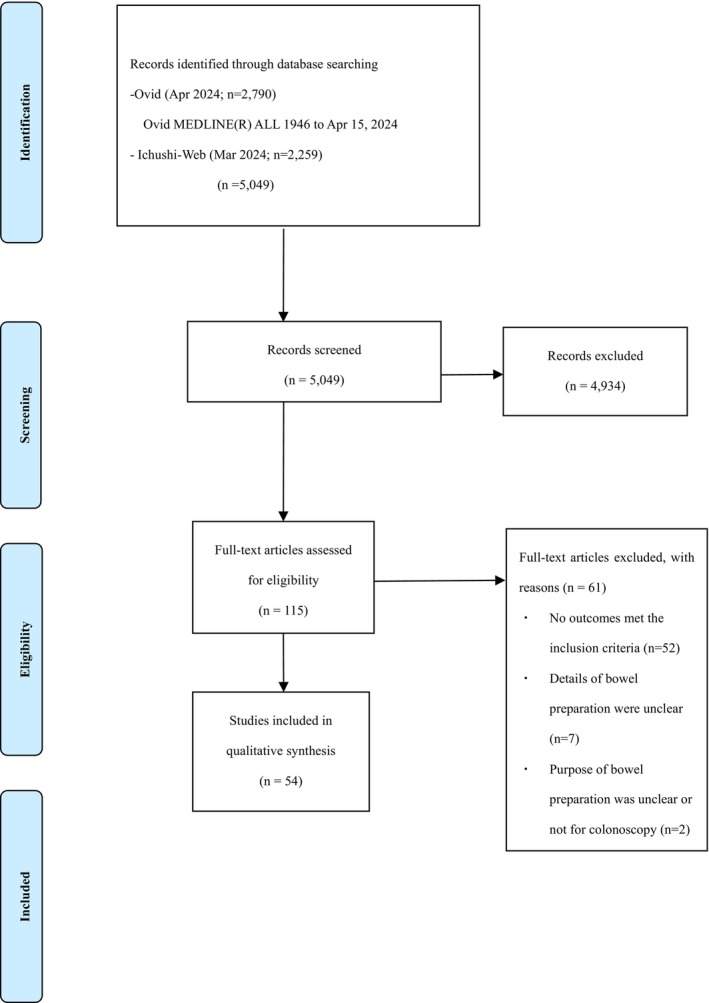
PRISMA flow diagram of study selection.

The flowchart of the study selection process is shown in Figure 1. The Ovid–MEDLINE search identified 2790 articles, and the Ichushi search identified 2259 articles, resulting in 5049 articles. The titles and abstracts of the publications were reviewed, and 115 articles were selected for full‐text evaluation. Ultimately, one case series study^29^ and 53 case reports^30^, ^31^, ^32^, ^33^, ^34^, ^35^, ^36^, ^37^, ^38^, ^39^, ^40^, ^41^, ^42^, ^43^, ^44^, ^45^, ^46^, ^47^, ^48^, ^49^, ^50^, ^51^, ^52^, ^53^, ^54^, ^55^, ^56^, ^57^, ^58^, ^59^, ^60^, ^61^, ^62^, ^63^, ^64^, ^65^, ^66^, ^67^, ^68^, ^69^, ^70^, ^71^, ^72^, ^73^, ^74^, ^75^, ^76^, ^77^, ^78^, ^79^, ^80^, ^81^, ^82^ were included in the analysis. The reasons for the exclusion of 61 articles were as follows: 52 did not report outcomes that met the inclusion criteria; seven had unclear details regarding bowel preparation (e.g. only mentioning “bowel preparation”); and two involved participants who were different from or unclear compared with the inclusion criteria.

### Risk of bias in included studies

One case series study was evaluated using the JBI Critical Appraisal Checklist for Case Series and received a score of 4/10. The study lacked clinical information about the participants, the outcomes or follow‐up results of cases, and the demographic information for the presenting sites. A total of 53 case reports were evaluated using the JBI Critical Appraisal Checklist for Case Reports, and 17 reports (33%) received a perfect score (8/8). The highest‐scoring criterion was clear reporting of diagnostic tests (Q4), with a score of 100% (43/43). The lowest score was for the criterion on medical history (Q2), with a score of 45.3% (24/53). Detailed assessments are provided in Appendix S2.

### Frequency of serious AEs

Only one study met the criteria for calculating the frequency of serious AEs.^29^ This study was a case series study assessing the frequency of bowel obstruction and perforation. A total of 86,463 colonoscopies conducted over a specific period were targeted, and AE cases were identified by searching clinical records. The frequency of bowel obstruction that met the inclusion criteria was 13.9 per 100,000 colonoscopies. Similarly, the frequency of bowel perforation was 2.3 per 100,000 colonoscopies. Serious AEs other than bowel obstruction and perforation were not included in the analysis.

### Patient and clinical characteristics of serious AEs

**Figure 2:**
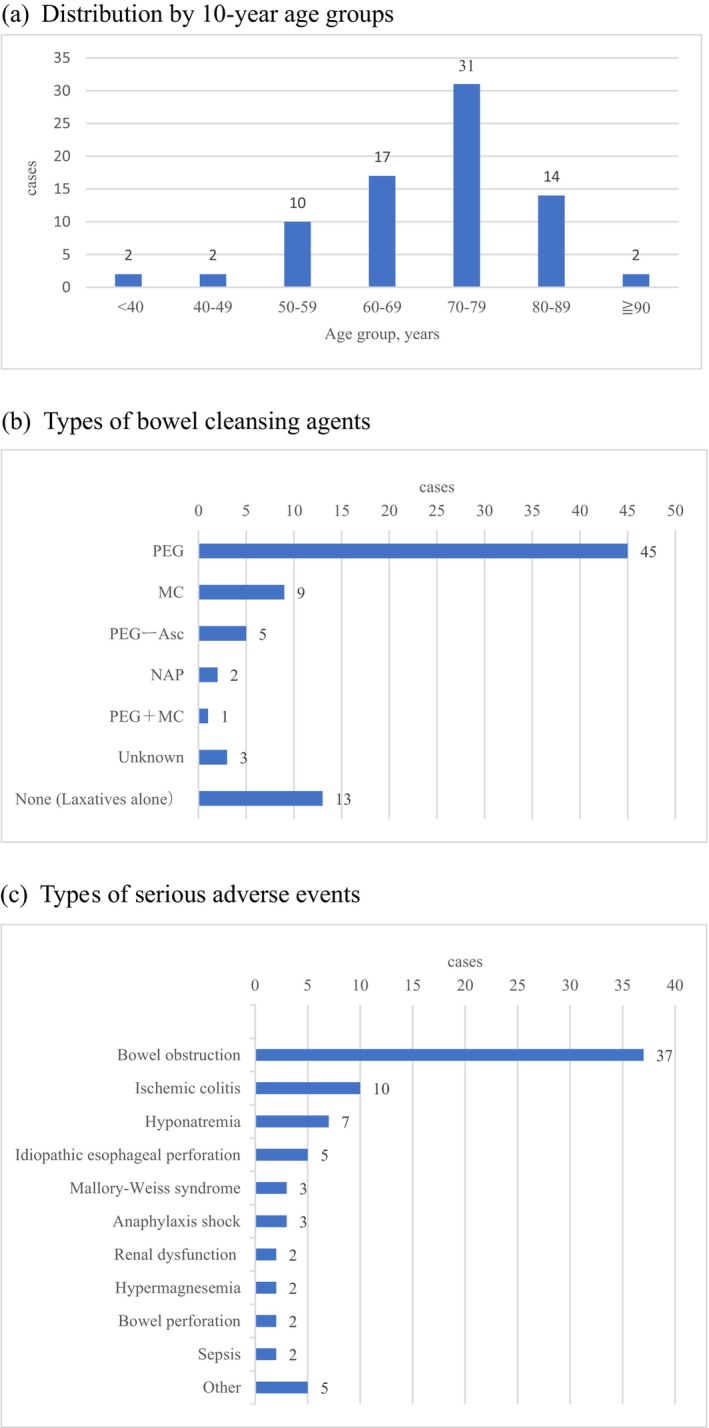
Patient and clinical characteristics of the 78 cases with serious adverse events. (a) Distribution of cases by 10‐year age groups. (b) Types of bowel‐cleansing agents used. (c) Types of serious adverse events observed. Asc, ascorbic acid; MC, magnesium citrate; NaP, sodium phosphate; PEG, polyethylene glycol electrolyte lavage solution.

Data from one case series study^29^ and 53 case reports^30^, ^31^, ^32^, ^33^, ^34^, ^35^, ^36^, ^37^, ^38^, ^39^, ^40^, ^41^, ^42^, ^43^, ^44^, ^45^, ^46^, ^47^, ^48^, ^49^, ^50^, ^51^, ^52^, ^53^, ^54^, ^55^, ^56^, ^57^, ^58^, ^59^, ^60^, ^61^, ^62^, ^63^, ^64^, ^65^, ^66^, ^67^, ^68^, ^69^, ^70^, ^71^, ^72^, ^73^, ^74^, ^75^, ^76^, ^77^, ^78^, ^79^, ^80^, ^81^, ^82^ were extracted, resulting in 78 patients who experienced serious AEs associated with bowel preparation for colonoscopy. The main results of these studies are summarized in Appendix S3. The publication years of the included articles ranged from 1995 to 2023. The most common age group was the 70s, followed by the 60s and 80s (Fig. 2a). Excluding seven cases with insufficient age information,^45^, ^51^, ^55^, ^65^, ^67^ the median age was 72.0 years (Table 1). The proportion of male patients was 59.0% (46/78), and 76.9% (60/78) had some comorbidity. The most common comorbidity was hypertension (26 cases), followed by a history of abdominal or pelvic surgery (20 cases) and heart disease (16 cases), with some cases having multiple comorbidities (Appendix S4).

**Table 1:** Patient characteristics of the 78 cases with serious adverse events

Patient characteristic	*N* = 78 cases
Age, years, median (IQR)^†^	72.0 (63.5–78.0)
Age group, years, *n* (%)	
<40	2 (2.6)
40–49	2 (2.6)
50–59	10 (12.8)
60–69	17 (21.8)
70–79	31 (39.7)
80–89	14 (17.9)
≥90	2 (2.6)
Sex, *n* (%)	
Male	46 (59.0)
Female	32 (41.0)
Comorbidity, *n* (%)	
Present	60 (76.9)
None	15 (19.2)
Unknown	3 (3.8)

^†^ Available in 71 cases.

IQR, interquartile range.

The primary purpose of colonoscopy was diagnosis in 60.3% (47/78) of cases. In colonoscopies performed for diagnostic purposes, symptomatic cases were the most common, at 50.0% (39/78), followed by positive FIT, at 7.7% (6/78). In symptomatic patients, constipation was the most commonly reported symptom. Primary screening accounted for 6.4% (5/78) of cases (Table 2a).

**Table 2:** Clinical characteristics of the 78 cases with serious adverse events (AEs)

Clinical characteristics *N* = 78 cases	*n* (%)
**(a) Purpose of colonoscopy and types of bowel preparation and agents**	
Purpose of colonoscopy	
Diagnostic test	47 (60.3)
Symptomatic individual	39 (50.0)
Constipation^†^	16
Abdominal pain^†^	11
Diarrhea or Soft stools^†^	5
Stool narrowing^†^	4
Lower gastrointestinal bleeding^†^	4
Anemia^†^	4
Abdominal distension^†^	3
Loss of appetite^†^	2
Weight loss^†^	2
Abnormal bowel movements^†^	1
FIT‐positive	6 (7.7)
Elevated tumor marker	2 (2.6)
Treatment or precise examination of tumor	14 (17.9)
Primary screening	5 (6.4)
Surveillance	2 (2.6)
Inflammatory bowel disease‐related	2 (2.6)
Unknown	8 (10.3)
**(b) Types of bowel preparation and agents**	
Types of bowel preparation	
Bowel‐cleansing agents and laxatives	30 (38.5)
Bowel‐cleansing agents alone	35 (44.9)
Laxatives alone	13 (16.7)
Types of bowel‐cleansing agents	
PEG	45 (57.7)
MC	9 (11.5)
PEG‐Asc	5 (6.4)
NaP	2 (2.6)
PEG+MC	1 (1.3)
Unknown	3 (3.8)
None (laxatives alone)	13 (16.7)
Timing of bowel‐cleansing agents administration	
Day of the colonoscopy	48 (61.5)
Day before the colonoscopy	3 (3.8)
Unknown	14 (17.9)
None (Laxatives alone)	13 (16.7)
**(c) Types and severity of AEs**	
Type of serious AEs	
Bowel obstruction	37 (47.4)
Ischemic colitis	10 (12.8)
Hyponatremia	7 (9.0)
Spontaneous esophageal rupture	5 (6.4)
Mallory—Weiss syndrome	3 (3.8)
Anaphylactic shock	3 (3.8)
Renal dysfunction	2 (2.6)
Hypermagnesemia	2 (2.6)
Bowel perforation	2 (2.6)
Sepsis	2 (2.6)
Other	5 (6.4)
Severity of AEs^‡^	
Grade 3	37 (47.4)
Grade 4	40 (51.3)
Grade 5	1 (1.3)

^†^ These data indicate that a single patient may have multiple entries.

^‡^ Classified according to the Common Terminology Criteria for Adverse Events (CTCAE) version 5.

Asc, ascorbic acid; FIT, fecal immunochemical test; MC, magnesium citrate; NaP, sodium phosphate; PEG, polyethylene glycol electrolyte lavage solution.

Regarding bowel preparation, bowel cleansing agents (alone or combined with laxatives) were used in 83.3% (65/78) of cases, whereas laxatives alone were used in 16.7% (13/78) of cases (Table 2b). The most common bowel cleansing agent was PEG, with various agents such as magnesium citrate (MC), PEG with ascorbic acid (PEG‐Asc), and sodium phosphate (NaP) also being used (Fig. 2b). Bowel preparation was usually done on the day of the examination.

Bowel obstruction was the most common type of AE, followed by ischemic colitis, hyponatremia, spontaneous esophageal rupture, Mallory–Weiss syndrome, and anaphylactic shock (Fig. 2c). The severity of AEs was Grade 3 in 47.4% (37/78), Grade 4 in 51.3% (40/78), and Grade 5 in 1.3% (1/78) of cases (Table 2c).

### Patient and clinical characteristics of AEs evaluated by age

Of all reported cases, 60.3% (47/78) were 70 years of age or older. The purpose of colonoscopy was primarily to diagnose the cause in symptomatic individuals, regardless of their age. Whereas primary screening purposes were not observed in the group aged 69 years or younger, they accounted for 10.6% (5/47) in the group aged 70 years or older. Furthermore, in the group aged 70 years or older, the proportion of Grade 4 or higher events was 59.6% (28/47), compared with 41.9% (13/31) in the group aged 69 years or younger, indicating a higher tendency in the older group (*P* = 0.166). In addition, there was one fatal case in the group aged 70 years or older (Table 3).

**Table 3:** Patient and clinical characteristics of cases with serious adverse events (AEs) by age group

	<69 years (*N* = 31 cases)	≥70 years (*N* = 47 cases)	*P*‐value^†^
	*n* (%)	*n* (%)	
Sex			0.244
Male	21 (67.7)	25 (53.2)	–
Female	10 (32.3)	22 (46.8)	–
Comorbidity			1
Present	24 (77.4)	36 (76.6)	–
None	6 (19.4)	9 (19.1)	–
Unknown	1 (3.2)	2 (4.3)	–
Purpose of colonoscopy			0.500
Diagnostic test^‡^	20 (64.5)	27 (57.4)	–
Symptomatic individual	19 (61.3)	20 (42.6)	–
FIT‐positive	1 (3.2)	5 (10.6)	–
Elevated tumor marker	0 (0.0)	2 (4.3)	–
Treatment or precise examination of tumor	5 (16.1)	9 (19.1)	–
Primary screening	0 (0.0)	5 (10.6)	–
Surveillance	1 (3.2)	1 (2.1)	–
Inflammatory bowel disease‐related	1 (3.2)	1 (2.1)	–
Unknown	4 (12.9)	4 (8.5)	–
Type of serious AEs			0.697
Bowel obstruction	16 (51.6)	21 (44.7)	–
Ischemic colitis	5 (16.1)	5 (10.6)	–
Hyponatremia	2 (6.5)	5 (10.6)	–
Spontaneous esophageal rupture	3 (9.7)	2 (4.3)	–
Mallory–Weiss syndrome	0 (0.0)	3 (6.4)	–
Anaphylactic shock	2 (6.5)	1 (2.1)	–
Renal dysfunction	1 (3.2)	1 (2.1)	–
Hypermagnesemia	0 (0.0)	2 (4.3)	–
Bowel perforation	0 (0.0)	2 (4.3)	–
Sepsis	1 (3.2)	1 (2.1)	–
Other	1 (3.2)	4 (8.5)	–
Severity of AEs^§^			0.166
Grade 3	18 (58.1)	19 (40.4)	–
Grade 4 or 5	13 (41.9)	28 (59.6)	–

^†^ *P*‐values were calculated using Fisher's exact test. This exploratory analysis included only patients with serious AEs and should be interpreted as a reference.

^‡^ The following were excluded from stratified analysis as diagnostic tests: symptomatic individuals, fecal immunochemical test (FIT)‐positive cases, and cases with elevated tumor markers.

^§^ Classified according to the Common Terminology Criteria for Adverse Events (CTCAE) version 5.

### Patient and clinical characteristics of AEs by purpose of colonoscopy

The purpose of colonoscopy differed among the age groups; 83.3% (5/6) of FIT‐positive cases were in their 70s and 100% (5/5) of primary screening cases in their 80s. The proportion of individuals with comorbidities was 83.3% (5/6) for FIT‐positive cases and 100% (5/5) for primary screening cases.

Bowel obstruction accounted for 69% (27/39) of AEs in diagnostic tests for symptomatic patients. In FIT‐positive cases, there were two cases each of bowel obstruction and hyponatremia and one case each of ischemic colitis and spontaneous esophageal rupture. In primary screening cases, there was one case each of bowel obstruction, ischemic colitis, spontaneous esophageal rupture, renal failure, and sepsis (Appendix S5).

### Patient and clinical characteristics by type of AE

The characteristics of each type of AE are shown in Tables 4 and 5 and Appendix S6. The most frequently reported AE was bowel obstruction, which occurred with several types of bowel‐cleansing agents, including PEG, as well as with laxatives alone. In those who developed bowel obstruction, 70.3% (26/37) of the chief complaints (purpose of the colonoscopy) were abdominal symptoms. Specific symptoms included abdominal pain, constipation, and diarrhea or loose stools, with some cases having multiple abdominal symptoms. The most common cause of bowel obstruction was cancer (70.3%), followed by fecal impaction (21.6%). Of the 26 cancer cases, 7.7% (2/26) of cancers occurred in the cecum and ascending colon, 11.5% (3/26) in the transverse colon, and 80.8% (21/26) in the rectum and sigmoid colon. In addition, all eight cases of fecal obstruction occurred in the sigmoid colon or rectum. Complications such as obstructive colitis, bowel perforation, hypermagnesemia, and sepsis were observed in 14 cases of bowel obstruction; of them, 93% (13/14) were aged 70 years or older. In the bowel obstruction cases, 51.3% (19/37) had a severity of Grade 4 or higher, including one Grade 5 case.

**Table 4:** Usage of bowel cleansing agents for each adverse event in the 78 cases

	PEG	MC	PEG‐Asc	NaP	PEG + MC	Unknown	None (laxatives alone)
Bowel obstruction (*N* = 37)	20	4	1	–	1	2	9
Ischemic colitis (*N* = 10)	4	1	1	–	–	–	4
Hyponatremia (*N* = 7)	4	1	2	–	–	–	–
Spontaneous esophageal rupture (*N* = 5)	4	–	–	–	–	1	–
Mallory–Weiss syndrome (*N* = 3)	3	–	–	–	–	–	–
Anaphylactic shock (*N* = 3)	3	–	–	–	–	–	–
Renal dysfunction (*N* = 2)	–	–	–	2	–	–	–
Hypermagnesemia (*N* = 2)	–	2 (3^†^)	–	–	–	–	(1^†^)
Bowel perforation (*N* = 2)	2	(1^†^)	–	–	–	–	(2^†^)
Sepsis (*N* = 2)	2	–	–	–	–	–	(1^†^)
Other (*N* = 5)	3	1	1	–	–	–	–
Total (*N* = 78)	45	9	5	2	1	3	13

^†^ The number of cases with bowel obstruction (the patient records are duplicated).

Asc, ascorbic acid; MC, magnesium citrate; NaP, sodium phosphate; PEG, polyethylene glycol electrolyte lavage solution.

**Table 5:** Patient and clinical characteristics of the 37 cases with bowel obstruction

Bowel obstruction *N* = 37 cases	*n* (%)
Chief complaint (purpose of colonoscopy)	
Abdominal symptom	26 (70.3)
Abdominal pain^†^	11
Constipation^†^	11
Diarrhea or soft stools^†^	5
Stool narrowing^†^	3
Lower gastrointestinal bleeding^†^	3
Abdominal distension^†^	3
Anemia only	1 (2.7)
No subjective symptoms^‡^	9 (24.3)
Unknown	1 (2.7)
Causal factors	
Cancer	26 (70.3)
Cecum, ascending colon	2 (5.4)
Transverse colon	3 (8.1)
Descending colon	0 (0)
Sigmoid colon	11 (29.7)
Rectum	10 (27.0)
Fecal obstruction in the sigmoid colon or rectum	8 (21.6)
Adhesion in the sigmoid colon	1 (2.7)
Stenosis of the transverse colon due to cholecystitis	1 (2.7)
Ileal intussusception	1 (2.7)
Complications	
Present	14 (37.8)
Obstructive colitis^†^	6
Sepsis or shock^†^	5
Hypermagnesemia^†^	4
Bowel perforation^†^	3
Aspiration pneumonia^†^	1
Nonocclusive mesenteric ischemia^†^	1
Portal venous gas^†^	1
Absent	23 (62.2)

^†^ These data indicate that a single patient may have multiple entries.

^‡^ Including fecal immunochemical test‐positive and others.

Ischemic colitis occurred in 40% (4/10) of cases with laxatives alone, and the severity was relatively lower, with only 10% (1/10) being Grade 4 or higher. All seven cases of hyponatremia were associated with bowel‐cleansing agents, with five cases in individuals aged 70 years or older and six cases in men. Anaphylaxis was associated with PEG, and renal impairment occurred with NaP. Hypermagnesemia, including cases associated with bowel obstruction, was observed in six cases, all involving women aged 70 years or older. Five of these cases involved using MC, and four cases were related to constipation treated with magnesium oxide.

## DISCUSSION

A specific case series study that reported the frequencies of serious AEs, including bowel obstruction and perforation, was identified. Of the 78 cases of serious AEs reported in 54 articles (primarily case reports), various types of AEs were triggered by taking bowel‐cleansing agents and laxatives. Most of these cases occurred in individuals aged 70 years or older (particularly those in their 70s), individuals with comorbidities, and during diagnostic tests (particularly in symptomatic individuals). Bowel obstruction was the most common serious AE, particularly frequent in patients with symptoms such as abdominal pain and constipation. Notably, a fatal case was reported due to bowel obstruction.

In the extracted case series study, proactive data collection using clinical records was conducted at a single Designated Cancer Care Hospital. The study found 13.9 cases of bowel obstruction and 2.3 cases of bowel perforation per 100,000 colonoscopies as serious AEs.^29^ As mentioned in the Introduction, few studies evaluated the frequencies of serious AEs. According to one of the few reports, a large‐scale survey conducted by the Japan Gastroenterological Endoscopy Society (JGES), found that the frequencies of bowel obstruction and bowel perforation related to bowel preparation for colonoscopy, excluding therapeutic procedures, were 1.2 and 0.1 per 100,000 colonoscopies, respectively.^20^, ^22^ This survey was conducted as a questionnaire of clinicians at multiple centers 2 years after the final intervention point, suggesting the possibility of underestimation.^13^ Subsequently, the survey method was changed to collect prospective data over 1 week as determined by each facility. AEs related to preparation for endoscopy, in general, increased ~26‐fold compared with the previous method (detailed results regarding bowel preparation for colonoscopy were not disclosed).^83^ Considering these findings, the differences in frequencies may be due to differences in study participants, but they might also be significantly affected by the methods used to investigate AEs.^84^

Seventy‐eight cases of serious AEs identified in the present study demonstrated several universal or specific characteristics that warrant attention. First, various bowel‐cleansing agents, primarily PEG, were used alone or in combination with laxatives. These bowel‐cleansing agents were associated with multiple serious AEs, including bowel obstruction, ischemic colitis, electrolyte abnormalities, spontaneous esophageal rupture, Mallory–Weiss syndrome, and anaphylaxis. They should be appropriately recognized. In addition, as previously reported,^7^, ^85^ there was a tendency to associate PEG‐related products with anaphylaxis, NaP with renal dysfunction, and MC with hypermagnesemia, necessitating continued caution. Furthermore, serious events such as bowel obstruction and ischemic colitis occurred even when only laxatives were taken before ingesting bowel‐cleansing agents, emphasizing the importance of recognizing this potential risk.

Next, attention should be paid to serious AEs primarily observed in individuals aged 70 years or older, those with comorbidities, and symptomatic patients, particularly bowel obstruction in patients with abdominal symptoms. The United States Preventive Services Task Force has pointed out that serious AEs are generally likely to depend on comorbid conditions.^5^ According to the Pharmaceuticals and Medical Devices Agency, there were 12 reported deaths related to bowel preparation for colonoscopy and other procedures, with 75% involving individuals aged 70 years or older and those with abdominal symptoms.^19^ The most common AEs were bowel obstruction or bowel perforation. In the aforementioned JGES survey, 80 cases related to bowel‐cleansing agents were reported, with bowel obstruction being the most frequent.^20^, ^22^ These study results support the present findings.

With aging, the incidences of chronic diseases and cancer increase, whereas immune function and physical function decline. In the present study, individuals aged 70 years or older tended to have more serious AEs than those under 70 years of age. In addition, complications related to bowel obstruction were more commonly reported in individuals aged 70 years and older. Furthermore, as in previous reports,^7^, ^85^ serious electrolyte abnormalities, such as hyponatremia and hypermagnesemia, were more common in individuals aged 70 years and over. Whereas these trends may not directly lead to an increase in mortality due to serious AEs, they suggest that elderly persons are at an overall higher risk.

A British systematic review reported that 131 AE cases related to bowel preparation used NaP or PEG.^6^ There were 15 fatal cases due to these AEs, 10 of which involved individuals aged 70 years and older, indicating a higher risk in elderly persons. In contrast, of the 22 AE cases associated with PEG, the most frequently reported events were Mallory–Weiss syndrome and spontaneous esophageal rupture, followed by electrolyte abnormalities. This suggests that the relatively large doses of 3–4 L of PEG may have contributed to these AEs. Furthermore, there was no mention of laxatives, and it is possible that laxatives were not used concurrently. These factors might explain the observed discrepancies. These results differ in trend from the findings of the present review.

Finally, the present study found that the number of AEs in colonoscopies performed for primary screening or due to positive FIT results was not particularly high compared with diagnostic purposes in symptomatic individuals. However, even for these purposes, various types of AEs still occur, and they are particularly notable in elderly persons and those with comorbidities. These results suggest that the risk of these serious AEs should be considered in primary screening and diagnostic evaluation of FIT‐positive cases. Specifically, the risk could be reduced by appropriately identifying symptomatic individuals and providing proper management for elderly persons and those with comorbidities.

Strategies for managing these severe AEs associated with bowel preparation have been outlined in international and domestic guidelines and recommendations regarding the appropriate administration of bowel preparations.^3^, ^4^, ^7^, ^19^ In particular, health‐care professionals involved in colonoscopy must recognize the potential for AEs associated with bowel preparation and understand the characteristics of AEs, as well as those of bowel‐cleansing agents and laxatives.^7^, ^19^ Subsequently, before administration, the risk of bowel preparation should be assessed individually for each patient through medical history assessment, including factors such as age, comorbidities, and bowel habits.^4^, ^19^ For suspected bowel stenosis during this process, a physical examination, abdominal radiography, or computed tomography is recommended to evaluate obstruction risk.^19^ Furthermore, based on these evaluations, it is necessary to individually determine the feasibility, type, and dosage of bowel‐cleansing agents or laxatives.^3^, ^4^, ^7^, ^19^ The findings of this review support the implementation of these guidelines and recommendations by providing practical information to enhance the safety of colonoscopy.

The strength of this study is that it is the first systematic review to elucidate the frequency and characteristics of serious AEs associated with bowel preparation for colonoscopy in Japan. This was achieved through a comprehensive evaluation of interventional and observational studies, including case reports.

This study has several limitations. First, the data obtained from this review reflect observational trends derived from case series and case reports, and they do not establish causal relationships or identify statistically significant risk factors. Although *P*‐values are presented in Table 3 to illustrate differences in proportions between age groups, these were calculated for reference only and should be interpreted with caution. Incorporating observational studies, such as case reports, into systematic reviews is important when evaluating rare AEs.^24^ Unless the sample is sufficiently large, even RCTs often fail to adequately capture the frequency of rare AEs.^6^, ^24^ In this review, no interventional studies met the inclusion criteria. Therefore, case reports and case series, although providing low‐certainty evidence, serve as valuable sources of information for understanding rare AEs and their patient characteristics by reflecting real‐world settings, and they contribute to hypothesis generation.^86^, ^87^ However, observational studies involve some bias that cannot be easily controlled; in particular, reporting bias must be considered in case reports and case series. Caution is required when interpreting their results, since they have the potential to either overestimate or underestimate. Second, only one study reported the frequency of bowel preparation. Although the present findings were compared with those of the JGES survey, the results were not directly comparable due to differences in context. Furthermore, a protocol for the present study was not registered, potentially raising concerns about transparency. To mitigate this, the PRISMA 2020 statement and necessary details were included in the text and Appendix S1.

Although major AEs related to bowel preparation were presented in this review, the information is insufficient to propose a plan for improvement. Further studies are needed to strengthen the national registry of AEs related to endoscopic examination, including the bowel preparation process.

## CONCLUSION

The frequency and characteristics of several serious AEs related to bowel preparation for colonoscopy in Japan were clarified. The present analysis may lead to more careful management of these serious AEs in clinical practice and CRC screening.

## CONFLICT OF INTERST

Authors declare no conflict of interest for this article.

## FUNDING INFORMATION

This work was supported by the National Cancer Center Research and Development Fund (grant number A‐21, 2023).

## Supporting information


**Appendix S1** Database search strategy.
**Appendix S2** The Joanna Briggs Institute (JBI) critical appraisal checklist for case series and case reports.
**Appendix S3** Overall results of 78 cases of serious adverse events related to bowel preparation for colonoscopy.
**Appendix S4** Details of comorbidities in the 78 cases.
**Appendix S5** Patient and clinical characteristics of cases with serious adverse events evaluated for the purpose of colonoscopy.
**Appendix S6** Patient and clinical characteristics of each adverse event in the 78 cases.
