# Supplementary material for: Serious adverse events associated with bowel preparation for colonoscopy in Japan: Systematic review
Source: Dig Endosc. 2025 Jun 5;37(9):905–18. doi: 10.1111/den.15055 (PMC12427159; doi:10.1111/den.15055)
Supplement: Supplementary file 1 — Appendix S1 Database search strategy. Appendix S2 The Joanna Briggs Institute (JBI) critical appraisal checklist for case series and case reports. Appendix S3 Overall results of 78 cases of serious adverse events related to bowel preparation for colonoscopy. Appendix S4 Details of comorbidities in the 78 cases. Appendix S5 Patient and clinical characteristics of cases with serious adverse events evaluated for the purpose of colonoscopy. Appendix S6 Patient and clinical characteristics of each adverse event in the 78 cases. [file DEN-37-905-s001.docx]

**Appendix S1.** Database search strategy: Strategy 1 for Ovid MEDLINE in English and strategy 2 for Ichushi-Web (the Japan Medical Abstracts Society) in Japanese.

Search strategy 1

Database: Ovid Medline

1 Colonoscopy/

2 TCS.ab,ti.

3 sigmoidoscopes/

4 (colonoscopy or colonoscopies or colonoscope).mp.

5 (sigmoidoscope or sigmoidoscopes or sigmoidoscopy or sigmoidoscopies).mp.

6 1 or 2 or 3 or 4 or 5

7 pretreatment*.ab,ti.

8 pre-treatment*.ab,ti.

9 prescreening*.ab,ti.

10 pre-screening*.ab,ti.

11 before colonoscop*.ab,ti.

12 prior colonoscop*.ab,ti.

13 before the colonoscop*.ab,ti.

14 cleansing*.ab,ti.

15 preparation*.ab,ti.

16 lavage*.ab,ti.

17 irrigat*.ab,ti.

18 7 or 8 or 9 or 10 or 11 or 12 or 13 or 14 or 15 or 16 or 17

19 accident*.ti,ab.

20 risk*.ti,ab.

21 effect*.ti,ab.

22 event*.ti,ab.

23 condition*.ti,ab.

24 reaction*.ti,ab.

25 19 or 20 or 21 or 22 or 23 or 24

26 6 and 18 and 25

Search strategy 2

Database: Ichushi-Web

#1 下剤/TH or 下剤/AL

#2 腸管洗浄剤/AL

#3 "Magnesium Citrate"/TH

#4 Golytely/TH

#5 Picoprep/TH

#6 Sulprep/TH

#7 "Sodium Phosphate"/TH

#8 腸管洗浄液/AL

#9 "Polyethylene Glycols"/TH or ポリエチレングリコール/AL

#10 MoviPrep/TH or モビプレップ/AL

#11 Golytely/TH or ムーベン/AL

#12 Golytely/TH or ニフレック/AL

#13 "Magnesium Oxide"/TH or 酸化マグネシウム/AL

#14 "Magnesium Citrate"/TH or マグコロール/AL

#15 "Sodium Phosphate"/TH or リン酸ナトリウム/AL

#16 ビジクリア/AL

#17 Sulprep/TH or サルプレップ/AL

#18 Picoprep/TH or ピコプレップ/AL

#19 前処置/AL

#20 ("腸洗浄"/TH or "intestinal lavage"/AL)

#21 "abdominal irrigation"/AL

#22 "bowel preparation"/AL

#23 "preparations"/AL

#24 "bowel cleansing"/AL

#25 "bowel-cleansing"/AL

#26 "colon cleansing"/AL

#27 "colonic cleansing"/AL

#28 "colonoscopy preparation"/AL

#29 "pretreatment"/AL

#30 "pre-treatment"/AL

#31 "pre-screening"/AL

#32 #1 or #2 or #3 or #4 or #5 or #6 or #7 or #8 or #9 or #10 or #11 or #12 or #13 or #14 or #15 or #16 or #17 or #18 or #19 or #20 or #21 or #22 or #23 or #24 or #25 or #26 or #27 or #28 or #29 or #30 or #31 "

#33 (内視鏡/TH or 内視鏡/AL)

#34 検査/AL

#35 大腸内視鏡/TH or 大腸内視鏡/AL

#36 下部内視鏡/AL

#37 S状結腸内視鏡法/TH or シグモイドスコピー/AL

#38 Colonoscopy/AL

#39 TCS/AL

#40 sigmoidoscopes/AL or sigmoidoscopy/AL or sigmoidoscopies/AL

#41 下部消化管内視鏡/AL

#42 #33 or #34 or #35 or #36 or #37 or #38 or #39 or #40 or #41

#43 (嘔吐/TH or 嘔吐/AL)

#44 おう吐/AL

#45 Vomiting/AL

#46 嘔気/AL

#47 吐き気/AL

#48 吐気/AL

#49 (腹痛/TH or 腹痛/AL)

#50 腹部痛/AL

#51 Abdominal/AL and Pain/AL

#52 (破裂/TH or 破裂/AL)

#53 特発性食道破裂/AL

#54 (アナフィラキシー/TH or アナフィラキシー/AL)

#55 (ショック/TH or ショック/AL)

#56 (腸穿孔/TH or 腸管穿孔/AL)

#57 イレウス/AL

#58 腸炎/TH

#59 閉塞性大腸炎/AL

#60 鼡径ヘルニア嵌頓/AL

#61 (水-電解質平衡異常/TH or 電解質異常/AL)

#62 (低ナトリウム血症/TH or 低ナトリウム血症/AL)

#63 低Na血症/AL

#64 (高マグネシウム血症/TH or 高マグネシウム血症/AL)

#65 高Mg血症/AL

#66 (低カルシウム血症/TH or 低カルシウム血症/AL)

#67 低Ca血症/AL

#68 (腎機能障害/TH or 腎不全/AL)

#69 急性リン酸腎症/AL

#70 虚血性腸炎/AL

#71 "Mallory-Weiss"/AL

#72 "マロリーワイス"/AL

#73 "マロリー・ワイス"/AL

#74 Rupture/AL

#75 Anaphylaxis/AL

#76 Shock/AL

#77 Perforation/AL

#78 偶発症/AL

#79 (合併症/TH or 合併症/AL)

#80 有害事象/AL

#81 副作用/AL

#82 ((腸閉塞/TH or 腸閉塞/AL or Obstruction/AL))

#83 #43 or #44 or #45 or #46 or #47 or #48 or #49 or #50 or #51 or #52 or #53 or #54 or #55 or #56 or #57 or #58 or #59 or #60 or #61 or #62 or #63 or #64 or #65 or #66 or #67 or #68 or #69 or #70 or #71 or #72 or #73 or #74 or #75 or #76 or #77 or #78 or #79 or #80 or #81 or #82

#84 #32 and #42 and #83

#85 ((#84) and (AB=Y and LA=日本語,英語 and CK=ヒト))

**Appendix S2.** The Joanna Briggs Institute (JBI) Critical Appraisal Checklist for Case Series and Case Reports

(a) JBI CHECKLIST FOR CASE SERIES

| Author, Year | 1. Were there clear criteria for inclusion in the case series? | 2. Was the condition measured in a standard, reliable way for all participants included in the case series? | 3. Were valid methods used for identification of the condition for all participants included in the case series? | 4. Did the case series have consecutive inclusion of participants? | 5. Did the case series have complete inclusion of participants? | 6. Was there clear reporting of the demographics of the participants in the study? | 7. Was there clear reporting of clinical information of the participants? | 8. Were the outcomes or follow up results of cases clearly reported? | 9. Was there clear reporting of the presenting site(s)/clinic(s) demographic information? | 10. Was statistical analysis appropriate? |
| --- | --- | --- | --- | --- | --- | --- | --- | --- | --- | --- |
| Yamauchi, 2017^29^ | Yes | Unclear | Unclear | Yes | Yes | Yes | No | No | No | Unclear |

(b) JBI CHECKLIST FOR CASE REPORTS

| Author, year | 1. Were patient’s demographic characteristics clearly described? | 2. Was the patient’s history clearly described and presented as a timeline? | 3. Was the current clinical condition of the patient on presentation clearly described? | 4. Were diagnostic tests or assessment methods and the results clearly described? | 5. Was the intervention(s) or treatment procedure(s) clearly described? | 6. Was the post-intervention clinical condition clearly described? | 7. Were adverse events (harms) or unanticipated events identified and described? | 8. Does the case report provide takeaway lessons? |
| --- | --- | --- | --- | --- | --- | --- | --- | --- |
| Saida, 1995^30^ | Yes | Yes | Yes | Yes | Yes | Yes | Yes | Yes |
| Tabata, 2002^31^ | Yes | Yes | Yes | Yes | Yes | No | No | Yes |
| Amamoto, 2004^32^ | Yes | Yes | Yes | Yes | Yes | Yes | Yes | Yes |
| Horiguchi, 2005^33^ | Yes | Yes | Yes | Yes | Yes | Yes | Unclear | Yes |
| Shimizu, 2005^34^ | Yes | No | Yes | Yes | Yes | No | No | Yes |
| Tsuda, 2006^35^ | Yes | No | Yes | Yes | Unclear | No | No | Yes |
| Ichimaru, 2009^36^ | Yes | No | Yes | Yes | Yes | Yes | Yes | Yes |
| Kikuchi, 2010^37^ | Yes | No | Yes | Yes | Yes | Yes | Yes | Yes |
| Kaida, 2014^38^ | Yes | Yes | Yes | Yes | Yes | Yes | Yes | Yes |
| Fukita, 2015^39^ | Yes | No | Yes | Yes | Yes | Yes | Yes | Yes |
| Miyauchi, 2016^40^ | Yes | No | Yes | Yes | Yes | Yes | Yes | Yes |
| Izuhara, 2016^41^ | Yes | Yes | Yes | Yes | Yes | No | Unclear | No |
| Uehira, 2018^42^ | Yes | No | Yes | Yes | Yes | Yes | Yes | Yes |
| Ishii, 2019^43^ | Yes | No | Yes | Yes | Yes | Yes | Yes | Yes |
| Nakamura, 2021^44^ | Yes | No | Yes | Yes | Yes | Yes | Unclear | Yes |
| Imai, 2022^45^ | Unclear | No | Yes | Yes | Yes | Yes | Unclear | Yes |
| Tomiki, 2001^46^ | Yes | No | Yes | Yes | Unclear | Yes | Yes | Yes |
| Munakata, 2002^47^ | Yes | No | Yes | Yes | Yes | Yes | Yes | Yes |
| Nakazawa, 2003^48^ | Yes | Yes | Yes | Yes | Yes | Yes | Yes | Yes |
| Takeyama, 2005^49^ | Yes | Yes | Yes | Yes | Yes | Yes | Yes | Yes |
| Yagami, 2012^50^ | Yes | No | Yes | Yes | Yes | Yes | Unclear | Yes |
| Matsuoka, 2014^51^ | Unclear | No | Yes | Yes | Yes | Yes | Yes | Yes |
| Inoue, 2014^52^ | Yes | Yes | Yes | Yes | Yes | Yes | Yes | Yes |
| Kusano, 2018^53^ | Yes | Yes | Yes | Yes | Yes | Yes | Unclear | Yes |
| Nagato, 2019^54^ | Yes | Yes | Yes | Yes | Yes | Yes | Yes | Yes |
| Wada, 2019^55^ | Unclear | No | Yes | Yes | Yes | Yes | Unclear | Unclear |
| Mashimo, 2003^56^ | Yes | Yes | Yes | Yes | Yes | Yes | Yes | Yes |
| Maeda, 2009^57^ | Yes | Yes | Yes | Yes | Yes | Yes | Yes | Yes |
| Katsuki, 2012^58^ | Yes | No | Yes | Yes | Yes | Yes | Yes | Yes |
| Nakajima, 2014^59^ | Yes | No | Yes | Yes | Yes | Yes | Yes | Yes |
| Yonemitsu, 2022^60^ | Yes | No | Yes | Yes | Yes | Yes | Yes | Yes |
| Nisida, 1999^61^ | Yes | Yes | Yes | Yes | Yes | Yes | Yes | Yes |
| Shimomura, 2003^62^ | Yes | No | Yes | Yes | Yes | Yes | Yes | Yes |
| Maruta, 2006^63^ | Yes | Yes | Yes | Yes | Yes | Yes | Unclear | Yes |
| Kakimoto, 2020^64^ | Yes | No | Yes | Yes | Yes | Yes | Unclear | Yes |
| Kawata, 2022^65^ | Unclear | No | Yes | Yes | Yes | Yes | Yes | Yes |
| Mathuura, 2022^66^ | Yes | Yes | Yes | Yes | Yes | Yes | Yes | Yes |
| Yamane, 2022^67^ | Unclear | Yes | Yes | Yes | Yes | Yes | Yes | Yes |
| Yamada, 2023^68^ | Yes | No | Yes | Yes | Yes | Yes | Unclear | Yes |
| Higuchi, 2013^69^ | Yes | No | Yes | Yes | Yes | Yes | Yes | Yes |
| Ouchi, 2019^70^ | Yes | Yes | Yes | Yes | Yes | Yes | Yes | Yes |
| Nakatsukasa, 2022^71^ | Yes | No | Yes | Yes | Yes | Yes | Yes | Yes |
| Yamazaki, 2016^72^ | Yes | Yes | Unclear | Yes | Yes | Yes | Yes | Yes |
| Imazu, 2022^73^ | Yes | No | Yes | Yes | Yes | Yes | Yes | Yes |
| Sugano, 2012^74^ | Yes | Yes | Yes | Yes | Yes | Yes | Yes | Yes |
| Yamada, 2016^75^ | Yes | No | Yes | Yes | Yes | Yes | Yes | Yes |
| Fukutomi, 2004^76^ | Yes | Yes | Yes | Yes | Yes | Yes | Yes | Yes |
| Suzuki, 2015^77^ | Yes | No | Yes | Yes | Yes | Yes | Yes | Yes |
| Nakaji, 2006^78^ | Yes | No | Yes | Yes | Yes | Yes | Yes | Yes |
| Kunoki, 2010^79^ | Yes | Yes | Yes | Yes | Yes | Yes | Yes | Yes |
| Nakazawa, 2016^80^ | Yes | Yes | Yes | Yes | Yes | Yes | Yes | Yes |
| Kohno, 2017^81^ | Yes | No | Yes | Yes | Yes | Yes | Yes | Yes |
| Imoto, 2018^82^ | Yes | Yes | Yes | Yes | Yes | Yes | Yes | Yes |

**Appendix S3.** Overall results of 78 cases of serious adverse events related to bowel preparation for colonoscopy

| No | Author, Year | Age (y) | Sex | Comorbidity | Indications for colonoscopy | Bowel cleansing agents (Timing of administration） | Use of laxatives | AEs | Main treatment | Severity of AEs^†^ | Terms used for severity in CTCAE v5.0 |
| --- | --- | --- | --- | --- | --- | --- | --- | --- | --- | --- | --- |
| 1 | Saida, 1995^30^ | 70 | F | None | Diarrhea, Abdominal distension | PEG (The day before the colonoscopy) | Yes | Bowel obstruction (Due to sigmoid colon cancer), Shock | Emergency surgery | Grade 4 | Colonic obstruction |
| 2 | Tabata, 2002^31^ | 77 | F | Hypertension | Constipation, Abdominal distension | PEG (Unkown) | Yes | Bowel obstruction (Due to rectal cancer), Obstructive colitis | Surgery | Grade 3 | Colonic obstruction |
| 3 | Amamoto, 2004^32^ | 57 | M | Diabetes mellitus, Myocardial infarction | Abdominal pain | None | Yes | Bowel obstruction (Due to sigmoid colon cancer), Bowel perforation, Portal venous gas | Emergency surgery | Grade 4 | Colonic obstruction |
| 4 | Horiguchi, 2005^33^ | 74 | M | COPD, Heart failure | FIT-positive | PEG  (The day of the colonoscopy) | Yes | Bowel obstruction (Due to feces) | Conservative treatment | Grade 3 | Colonic obstruction |
| 5 | Horiguchi, 2005 | 63 | M | Pulmonary tuberculosis | Constipation, Abdominal pain | PEG (The day of the colonoscopy) | Yes | Bowel obstruction (Due to rectal cancer) | Transanal drainage tube, Surgery | Grade 3 | Colonic obstruction |
| 6 | Shimizu, 2005^34^ | 76 | F | None | Melena | Bowel cleansing agent (Unkown) | Yes | Bowel obstruction (Due to rectal cancer), Obstructive colitis | Surgery | Grade 3 | Colonic obstruction |
| 7 | Tsuda, 2006^35^ | 76 | M | Unknown | Elevated tumor marker | None | Yes | Bowel obstruction (Due to transverse colon cancer) | Surgery | Grade 3 | Colonic obstruction |
| 8 | Ichimaru, 2009^36^ | 82 | M | Hypertension, Bronchial asthma | Constipation, Abdominal pain | Bowel cleansing agent (The day of the colonoscopy) | None | Bowel obstruction (Due to feces) | Conservative treatment | Grade 3 | Colonic obstruction |
| 9 | Kikuchi, 2010^37^ | 79 | F | After total hysterectomy, Polymyositis, Constipation (Taking oral magnesium oxide) | FIT-positive | MC (The day before the colonoscopy) | None | Bowel obstruction (Due to adhesion), Bowel perforation, Hypermagnesemia, Shock | Treatment in ICU, Conservative treatment | Grade 4 | Colonic obstruction |
| 10 | Kaida, 2014^38^ | 75 | F | Hypertension | Soft stools | None | Yes | Bowel obstruction (Due to rectal cancer), Bowel perforation, Shock | Emergency surgery | Grade 4 | Colonic obstruction |
| 11 | Fukita, 2015^39^ | 71 | M | Post-cholecystectomy | Hematochezia | PEG (The day of the colonoscopy) | Yes | Bowel obstruction (Due to rectal cancer), Obstructive colitis | Surgery | Grade 3 | Colonic obstruction |
| 12 | Fukita, 2015 | 73 | M | Diabetes mellitus, Glomerulosclerosis | Treatment for tumor | PEG (The day of the colonoscopy) | Yes | Bowel obstruction (Due to feces), Obstructive colitis | Conservative treatment | Grade 3 | Colonic obstruction |
| 13 | Miyauchi, 2016^40^ | 82 | F | Hypertension, Hyperlipidemia, Angina pectoris, (Taking oral magnesium oxide) | Unknown | MC (The day before the colonoscopy) | Yes | Bowel obstruction (Due to feces), Obstructive colitis, Hypermagnesemia | Treatment in ICU, Conservative treatment | Grade 4 | Colonic obstruction |
| 14 | Izuhara, 2016^41^ | 53 | F | After total hysterectomy | Diarrhea | None | Yes | Bowel obstruction (Due to rectal cancer) | Transanal drainage tube, surgery | Grade 3 | Colonic obstruction |
| 15 | Yamauchi, 2017^29^ | 65 | M | None | Constipation | PEG (The day of the colonoscopy) | None | Bowel obstruction (Due to ascending colon cancer) | Long intestinal tube, surgery | Grade 3 | Colonic obstruction |
| 16 | Yamauchi, 2017 | 62 | M | None | Stool narrowing, Abdominal pain | PEG (The day of the colonoscopy) | Yes | Bowel obstruction (Due to ascending colon cancer) | Long intestinal tube, emergency surgery | Grade 4 | Colonic obstruction |
| 17 | Yamauchi, 2017 | 70 | M | Diabetes mellitus, Hypertension | Stool narrowing | PEG (The day of the colonoscopy) | None | Bowel perforation (Due to rectal cancer) | Emergency surgery | Grade 4 | Colonic perforation |
| 18 | Yamauchi, 2017 | 62 | M | Colon diverticulum, Post-appendectomy | Constipation | None | Yes | Bowel obstruction (Due to rectal cancer) | Long intestinal tube, emergency surgery | Grade 4 | Colonic obstruction |
| 19 | Yamauchi, 2017 | 81 | F | None | Constipation, Hematochezia | None | Yes | Bowel obstruction (Due to sigmoid colon cancer) | Surgery | Grade 3 | Colonic obstruction |
| 20 | Yamauchi, 2017 | 91 | M | Hypertension, Hyperuricemia, Dementia, Post-cholecystectomy | Constipation, Abdominal pain | MC (The day of the colonoscopy) | Yes | Bowel obstruction (Due to transverse colon cancer) | Long intestinal tube, emergency surgery | Grade 4 | Colonic obstruction |
| 21 | Yamauchi, 2017 | 39 | F | None | Abdominal pain, Jaundice | PEG (The day of the colonoscopy) | None | Bowel obstruction (Due to sigmoid colon cancer) | Surgery | Grade 3 | Colonic obstruction |
| 22 | Yamauchi, 2017 | 69 | M | Unknown | Abdominal pain | PEG (The day of the colonoscopy) | None | Bowel obstruction (Stenosis due to cholecystitis) | Long intestinal tube, surgery | Grade 3 | Colonic obstruction |
| 23 | Yamauchi, 2017 | 71 | F | Unknown | Unknown (Asymptomatic individual) | PEG (The day of the colonoscopy) | Yes | Bowel obstruction (Due to feces) | Conservative treatment | Grade 3 | Colonic obstruction |
| 24 | Yamauchi, 2017 | 71 | F | None | Unknown (Asymptomatic individual) | PEG (The day of the colonoscopy) | Yes | Bowel perforation (Due to sigmoid colon cancer) | Emergency surgery | Grade 4 | Colonic perforation |
| 25 | Yamauchi, 2017 | 72 | M | Cerebral infarction, Diabetes mellitus, Hypertension, Atrial fibrillation, Lung cancer | Unknown (Asymptomatic individual) | PEG (The day of the colonoscopy) | Yes | Bowel obstruction (Due to transverse colon cancer) | Long intestinal tube, emergency surgery | Grade 4 | Colonic obstruction |
| 26 | Yamauchi, 2017 | 68 | F | Diabetes mellitus, Angina pectoris, Hyperlipidemia, Post-appendectomy | Constipation | PEG, MC (The day of the colonoscopy) | Yes | Bowel obstruction (Due to rectal cancer) | Surgery | Grade 3 | Colonic obstruction |
| 27 | Yamauchi, 2017 | 68 | M | Myocardial infarction, Post-appendectomy | Diarrhea | PEG (The day of the colonoscopy) | Yes | Bowel obstruction (Due to sigmoid colon cancer) | Endoscopic stenting, surgery | Grade 3 | Colonic obstruction |
| 28 | Yamauchi, 2017 | 57 | M | Post-appendectomy | Stool narrowing, Abdominal pain | PEG (The day of the colonoscopy) | None | Bowel obstruction (Due to sigmoid colon cancer) | Endoscopic stenting, surgery | Grade 3 | Colonic obstruction |
| 29 | Yamauchi, 2017 | 57 | M | Heart failure, Post-appendectomy | Constipation | PEG (The day of the colonoscopy) | Yes | Bowel obstruction (Due to rectal cancer) | Emergency surgery | Grade 4 | Colonic obstruction |
| 30 | Yamauchi, 2017 | 61 | M | Colon diverticulum | Diarrhea, Abdominal pain | PEG (The day of the colonoscopy) | Yes | Bowel obstruction (Due to sigmoid colon cancer) | Emergency surgery | Grade 4 | Colonic obstruction |
| 31 | Yamauchi, 2017 | 35 | F | Gastric ulcer | Constipation | PEG (The day of the colonoscopy) | Yes | Bowel obstruction (Due to sigmoid colon cancer) | Emergency surgery | Grade 4 | Colonic obstruction |
| 32 | Yamauchi, 2017 | 69 | F | Post-uterine prolapse surgery | Abdominal pain | None | Yes | Bowel obstruction (Due to sigmoid colon cancer) | Long intestinal tube, surgery | Grade 3 | Colonic obstruction |
| 33 | Yamauchi, 2017 | 70 | M | None | Constipation, Abdominal distension | PEG (The day of the colonoscopy) | None | Bowel obstruction (Due to sigmoid colon cancer) | Emergency surgery | Grade 4 | Colonic obstruction |
| 34 | Yamauchi, 2017 | 60 | M | Colon diverticulum | Stool narrowing | PEG (The day of the colonoscopy) | None | Bowel obstruction (Due to sigmoid colon cancer) | Emergency surgery | Grade 4 | Colonic obstruction |
| 35 | Uehira, 2018^42^ | 90 | M | Hypertension, Diabetes mellitus, MDS | Anemia | PEG (Unkown) | None | Bowel obstruction (Due to intussusception), Aspiration pneumonia | Treatment in ICU, Emergency surgery | Grade 4 | Colonic obstruction |
| 36 | Ishii, 2019^43^ | 82 | F | Hypertension, Atrial fibrillation, Mild chronic renal failure | Primary screening | PEG-Asc (Unkown) | Yes | Bowel obstruction (Due to feces), Non-occlusive mesenteric ischemia, Shock | Treatment in ICU, Emergency surgery | Grade 4 | Colonic obstruction |
| 37 | Nakamura, 2021^44^ | 81 | F | Hypertension, Post-appendectomy, (Taking oral magnesium oxide) | Precise examination of rectal cancer | None | Yes | Bowel obstruction (Due to rectal cancer), Obstructive colitis, Hypermagnesemia | Treatment in ICU, Surgery | Grade 4 | Colonic obstruction |
| 38 | Imai, 2022^45^ | 70s | F | Colonic adenoma, Colonic diverticulum | Abdominal pain | None | Yes | Bowel obstruction (Due to rectal cancer), Obstructive colitis, Hypermagnesemia | Emergency surgery | Grade 5 | Colonic obstruction |
| 39 | Tomiki, 2001^46^ | 60 | M | Post-surgery for rectal cancer | Postoperative surveillance | None | Yes | Ischemic colitis | Conservative treatment | Grade 3 | Colonic hemorrhage |
| 40 | Munakata, 2002^47^ | 72 | M | None | Treatment for tumor | PEG (The day of the colonoscopy) | Yes | Ischemic colitis | Conservative treatment | Grade 3 | Colonic hemorrhage |
| 41 | Nakazawa, 2003^48^ | 44 | M | Hemorrhoids | Anal bleeding, Anemia | PEG (The day of the colonoscopy) | None | Ischemic colitis | Conservative treatment | Grade 3 | Colonic hemorrhage |
| 42 | Takeyama, 2005^49^ | 78 | M | Post-appendectomy | Precise examination of rectal cancer | PEG (The day of the colonoscopy) | Yes | Ischemic proctitis | Conservative treatment | Grade 3 | Colonic hemorrhage |
| 43 | Yagami, 2012^50^ | 85 | F | Hypertension, Hydrocephalus | Elevated tumor marker | MC (The day of the colonoscopy) | Yes | Ischemic colitis | Conservative treatment | Grade 3 | Colonic hemorrhage |
| 44 | Matsuoka, 2014^51^ | 50s | F | Hypertension, Post-uterine fibroid surgery | FIT-positive | None | Yes | Ischemic colitis | Conservative treatment | Grade 3 | Colonic hemorrhage |
| 45 | Inoue, 2014^52^ | 85 | F | Hypertension, Atrial fibrillation, thyroid cancer, Ischemic colitis | Primary screening | None | Yes | Ischemic colitis | Emergency surgery | Grade 4 | Colonic hemorrhage |
| 46 | Kusano, 2018^53^ | 64 | F | Hypertension, Depression | Constipation | PEG (The day of the colonoscopy) | Yes | Ischemic colitis | Conservative treatment | Grade 3 | Colonic hemorrhage |
| 47 | Nagato, 2019^54^ | 78 | F | Post-surgery for rectal cancer | Postoperative surveillance | None | Yes | Ischemic colitis | Conservative treatment | Grade 3 | Colonic hemorrhage |
| 48 | Wada, 2019^55^ | 60s | F | None | Treatment for tumor | PEG-Asc (Unkown) | Yes | Ischemic colitis | Conservative treatment | Grade 3 | Colonic hemorrhage |
| 49 | Mashimo, 2003^56^ | 76 | F | Hiatal hernia, Reflux esophagitis, Rapidly progressive glomerulonephritis | FIT-positive | PEG (Unkown) | None | Spontaneous esophageal rupture | Conservative treatment | Grade 3 | Esophageal perforation |
| 50 | Maeda, 2009^57^ | 52 | F | None | Constipation | PEG (Unkown) | None | Spontaneous esophageal rupture | Emergency surgery | Grade 4 | Esophageal perforation |
| 51 | Katsuki, 2012^58^ | 51 | M | Alcoholic liver cirrhosis, Bipolar disorder | Unknown | PEG (Unkown) | None | Spontaneous esophageal rupture | Emergency surgery | Grade 4 | Esophageal perforation |
| 52 | Nakajima, 2014^59^ | 80 | M | Rheumatoid arthritis, Arrhythmia | Primary screening | PEG (The day of the colonoscopy) | None | Spontaneous esophageal rupture | Conservative treatment | Grade 3 | Esophageal perforation |
| 53 | Yonemitsu, 2022^60^ | 69 | F | Myasthenia gravis, Good syndrome | Unknown | Bowel cleansing agent (Unkown) | None | Spontaneous esophageal rupture | Emergency surgery | Grade 4 | Esophageal perforation |
| 54 | Nishida, 1999^61^ | 76 | F | None | Precise examination of tumor | PEG (Unkown) | None | Mallory-Weiss syndrome | Emergency surgery | Grade 4 | Upper gastrointestinal hemorrhage |
| 55 | Shimomura, 2003^62^ | 77 | M | Cerebral infarction | Loss of appetite, Fatigue | PEG (The day of the colonoscopy) | None | Mallory-Weiss syndrome | Endoscopic hemostasis | Grade 4 | Upper gastrointestinal hemorrhage |
| 56 | Maruta, 2006^63^ | 81 | M | Hypertension, Angina pectoris | Abnormal bowel movements | PEG (The day of the colonoscopy) | None | Mallory-Weiss syndrome | Endoscopic hemostasis | Grade 4 | Upper gastrointestinal hemorrhage |
| 57 | Kakimoto, 2020^64^ | 74 | M | None | FIT-positive | MC (The day of the colonoscopy) | None | Hyponatremia (Na 119 mg/dl) | Medical treatment | Grade 4 | Hyponatremia |
| 58 | Kawata, 2022^65^ | 70s | M | Hypertension | Treatment for tumor | PEG (The day of the colonoscopy) | Yes | Hyponatremia (Na 122 mg/dl) | Medical treatment | Grade 3 | Hyponatremia |
| 59 | Kawata, 2022 | 70s | M | Cardiac pacemaker, Hypertension | FIT-positive | PEG (The day of the colonoscopy) | Yes | Hyponatremia (Na 107 mg/dl) | Medical treatment | Grade 4 | Hyponatremia |
| 60 | Matsuura, 2022^66^ | 71 | F | Hypothyroidism | Loss of appetite | PEG-Asc (The day of the colonoscopy) | None | Hyponatremia (Na 121 mg/dl) | Medical treatment | Grade 3 | Hyponatremia |
| 61 | Yamane, 2022^67^ | 60s | M | Gastric ulcer (Post-gastrectomy), Reflux esophagitis | Treatment for tumor | PEG (The day of the colonoscopy) | None | Hyponatremia (Na 120 mg/dl) | Medical treatment | Grade 3 | Hyponatremia |
| 62 | Yamane, 2022 | 50s | M | None | Treatment for tumor | PEG (The day of the colonoscopy) | None | Hyponatremia (Na 124 mg/dl) | Medical treatment | Grade 3 | Hyponatremia |
| 63 | Yamada, 2023^68^ | 86 | M | Atrial fibrillation, Heart failure, Hypertension, Diabetes mellitus, Asthma | Anemia, Weight loss | PEG-Asc (The day of the colonoscopy) | None | Hyponatremia (Na 114 mg/dl) | Medical treatment | Grade 4 | Hyponatremia |
| 64 | Higuchi, 2013^69^ | 75 | M | Gastric cancer (Post-gastrectomy) | Constipation, Weight loss | PEG (The day of the colonoscopy) | Yes | Anaphylactic shock | Adrenaline injection | Grade 4 | Anaphylaxis |
| 65 | Ouchi, 2019^70^ | 44 | M | Urticaria and anaphylaxis due to food | Treatment for tumor | PEG (The day of the colonoscopy) | None | Anaphylactic shock | Adrenaline injection | Grade 4 | Anaphylaxis |
| 66 | Nakatsukasa, 2022^71^ | 59 | M | Diabetes mellitus, Heart failure, Myocardial infarction | Unknown | PEG (The day of the colonoscopy) | None | Anaphylactic shock | Adrenaline injection | Grade 4 | Anaphylaxis |
| 67 | Yamazaki, 2016^72^ | 85 | F | Hypertension, Constipation (Taking oral magnesium oxide) | Constipation | MC (The day of the colonoscopy) | None | Hypermagnesemia (Mg 11.0 mg/dl) | Treatment in ICU, Medical treatment | Grade 4 | Hypermagnesemia |
| 68 | Imazu, 2022^73^ | 77 | F | Post-surgery for colon cancer, Hyperlipidemia | Treatment for tumor | MC (Unkown) | None | Bowel obstruction (Due to feces), Hypermagnesemia (Mg 13.7 mg/dl) | Conservative treatment | Grade 4 | Hypermagnesemia |
| 69 | Imazu, 2022 | 70 | F | Stenosis post-surgery for colon cancer, Parkinson's disease (Taking oral magnesium oxide) | Constipation | MC (Unkown) | None | Hypermagnesemia (Mg 10.6 mg/dl) | Balloon dilation, Medical treatment | Grade 4 | Hypermagnesemia |
| 70 | Sugano, 2012^74^ | 69 | M | Hypertension, Hyperlipidemia | Precise examination of colon cancer | NAP (The day of the colonoscopy) | None | Renal dysfunction | Medical treatment | Grade 3 | Acute kidney injury |
| 71 | Yamada, 2016^75^ | 86 | F | CKD, Hypertension, Hyperlipidemia, Osteoarthritis of the knee | Primary screening | NAP (Unkown) | None | Renal dysfunction (Acute phosphate nephropathy) | Medical treatment | Grade 3 | Acute kidney injury |
| 72 | Fukutomi, 2004^76^ | 58 | M | Ulcerative Colitis, Myocardial infarction | Follow-up for ulcerative Colitis | PEG (The day of the colonoscopy) | None | Sepsis,  Intra vertebral canal abscess | Medical treatment, Surgical drainage for the abscess. | Grade 3 | Sepsis |
| 73 | Suzuki, 2015^77^ | 82 | M | Gastric cancer, Hypertension, Hyperlipidemia, Post-coronary artery bypass surgery, Post-abdominal aortic aneurysm surgery | Primary screening | PEG (The day of the colonoscopy) | None | Septic shock, DIC | Medical treatment | Grade 4 | Sepsis |
| 74 | Nakaji, 2006^78^ | 64 | M | Chronic hepatitis C, Chronic pancreatitis, Hypertension | Unknown | PEG (The day of the colonoscopy) | Yes | Reduction of left inguinal hernia | Manual reduction | Grade 4 | General principles |
| 75 | Kunoki, 2010^79^ | 75 | M | Hypertension, Diabetes mellitus, Cerebral infarction, Post-appendectomy | Treatment for tumor | PEG (The day of the colonoscopy) | None | Diverticular bleeding | Endoscopic hemostasis | Grade 4 | Lower gastrointestinal hemorrhage |
| 76 | Nakazawa, 2016^80^ | 75 | M | Ulcerative colitis | Recurrent ulcerative colitis | MC (The day of the colonoscopy) | Yes | Sigmoid volvulus | Endoscopic detorsion | Grade 4 | General principles |
| 77 | Kohno, 2017^81^ | 77 | M | None | Treatment for tumor | PEG (Unkown) | None | Crowned Dens Syndrome | Medical treatment | Grade 3 | General principles |
| 78 | Imoto, 2018^82^ | 82 | M | Hypertension | Anemia | PEG-Asc (The day of the colonoscopy) | Yes | Acute myocardial infarction | Percutaneous coronary intervention | Grade 4 | Myocardial infarction |

AE, Adverse event; PEG, Polyethylene glycol electrolyte lavage solution; PEG-Asc, PEG with ascorbic acid; MC, Magnesium citrate; NaP, Sodium phosphate.

^†^Classified according to CTCAE version 5

**Appendix S4.** Details of comorbidities in the 78 cases

|  |  |
| --- | --- |
| Comorbidity^†^ | n |
| Hypertension | 26 |
| Post-abdominal and pelvic surgery | 20 |
| Heart disease | 16 |
| Diabetes mellitus | 8 |
| Hyperlipidemia | 6 |
| Neurological disorder | 5 |
| Respiratory disease | 5 |
| Renal disease | 4 |
| Colon diverticulum | 4 |
| Autoimmune disease | 3 |
| Constipation | 2 |
| Psychiatric disorder | 2 |
| Ulcerative colitis | 2 |
| Other | 13 |

^†^These data indicate that a single patient may have multiple entries.

**Appendix S5.** Patient and clinical characteristics of cases with serious adverse events evaluated by the purpose of colonoscopy

|  |  | Symptomatic individual N = 39 cases | | FIT-positive N = 6 cases | | Primary screening N = 5 cases | |
| --- | --- | --- | --- | --- | --- | --- | --- |
| Sex | | n | (%) | n | (%) | n | (%) |
|  | Male | 23 | (59.0) | 3 | (50.0) | 3 | (60.0) |
| Age group, y, | |  |  |  |  |  |  |
|  | <40 | 2 | (5.1) |  |  |  |  |
|  | 40-49 | 1 | (2.6) |  |  |  |  |
|  | 50-59 | 5 | (12.8) | 1 | (16.7) |  |  |
|  | 60-69 | 11 | (28.2) |  |  |  |  |
|  | 70-79 | 12 | (30.8) | 5 | (83.3) |  |  |
|  | 80-89 | 6 | (15.4) |  |  | 5 | (100) |
|  | ≥90 | 2 | (5.1) |  |  |  |  |
| Comorbidity | |  |  |  |  |  |  |
|  | Present | 30 | (76.9) | 5 | (83.3) | 5 | (100) |
|  | None | 8 | (20.5) | 1 | (16.7) |  |  |
|  | Unknown | 1 | (2.6) |  |  |  |  |
| Types of bowel preparation | |  |  |  |  |  |  |
|  | Bowel cleansing agents and laxatives | 15 | (38.5) | 2 | (33.3) | 1 | (20.0) |
|  | Bowel cleansing agents alone | 17 | (43.6) | 3 | (50.0) | 3 | (60.0) |
|  | Laxatives alone | 7 | (17.9) |  |  | 1 | (20.0) |
| Types of serious AEs | |  |  |  |  |  |  |
|  | Bowel obstruction | 27 | (69.2) | 2 | (33.3) | 1 | (20.0) |
|  | Ischemic colitis | 2 | (5.1) | 1 | (16.7) | 1 | (20.0) |
|  | Hyponatremia | 2 | (5.1) | 2 | (33.3) |  |  |
|  | Spontaneous esophageal rupture | 1 | (2.6) | 1 | (16.7) | 1 | (20.0) |
|  | Mallory-Weiss syndrome | 2 | (5.1) |  |  |  |  |
|  | Anaphylactic shock | 1 | (2.6) |  |  |  |  |
|  | Renal dysfunction |  |  |  |  | 1 | (20.0) |
|  | Hypermagnesemia | 2 | (5.1) |  |  |  |  |
|  | Bowel perforation | 1 | (2.6) |  |  |  |  |
|  | Sepsis |  |  |  |  | 1 | (20.0) |
|  | Other | 1 | (2.6) |  |  |  |  |
| Severity of AEs^†^ | |  |  |  |  |  |  |
|  | Grade 3 | 17 | (43.6) | 3 | (50.0) | 2 | (40.0) |
|  | Grade 4 | 21 | (53.8) | 3 | (50.0) | 3 | (60.0) |
|  | Grade 5 | 1 | (2.6) |  |  |  |  |

AE, Adverse event.

^†^Classified according to CTCAE version 5

**Appendix S6.** Patient and clinical characteristics of each adverse event in the 78 cases

|  | Age ≥70 years | | | | Male (Sex) | | | | | Symptomatic individual  (Purpose of colonoscopy) | | | | | ≥Grade 4  (Severity of AE) ^†^ | | | | |
| --- | --- | --- | --- | --- | --- | --- | --- | --- | --- | --- | --- | --- | --- | --- | --- | --- | --- | --- | --- |
|  | n/N | (%) | n/N | (%) | | n/N | (%) | n/N | (%) | | n/N | (%) | n/N | (%) | | n/N | (%) | n/N | (%) |
| Bowel obstruction (N=37) | 21/37 | (57) |  |  | | 21/37 | (57) |  |  | | 27/37 | (73) |  |  | | 19/37 | (51) |  |  |
| Ischemic colitis (N=10) | 5/10 | (50) |  |  | | 4/10 | (40) |  |  | | 2/10 | (20) |  |  | | 1/10 | (10) |  |  |
| Hyponatremia (N=7) | 5/7 | (71) |  |  | | 6/7 | (86) |  |  | | 2/7 | (29) |  |  | | 3/7 | (43) |  |  |
| Spontaneous esophageal  rupture (N=5) | 2/5 | (40) |  |  | | 2/5 | (40) |  |  | | 1/5 | (20) |  |  | | 3/5 | (60) |  |  |
| Mallory-Weiss syndrome (N=3) | 3/3 | (100) |  |  | | 2/3 | (67) |  |  | | 2/3 | (67) |  |  | | 3/3 | (100) |  |  |
| Anaphylactic shock (N=3) | 1/3 | (33) |  |  | | 3/3 | (100) |  |  | | 1/3 | (33) |  |  | | 3/3 | (100) |  |  |
| Renal dysfunction (N=2) | 1/2 | (50) |  |  | | 1/2 | (50) |  |  | | 0/2 | (0) |  |  | | 0/2 | (0) |  |  |
| Hypermagnesemia (N=2) | 2/2 | (100) | 6/6^‡^ | (100) | | 0/2 | (0) | 0/6^‡^ | (0) | | 2/2 | (100) | 2/6^‡^ | (33) | | 2/2 | (100) | 6/6^‡^ | (100) |
| Bowel perforation (N=2) | 2/2 | (100) | 4/5^‡^ | (80) | | 1/2 | (50) | 2/5^‡^ | (40) | | 1/2 | (50) | 3/5^‡^ | (60) | | 2/2 | (100) | 5/5^‡^ | (100) |
| Sepsis (N=2) | 1/2 | (50) | 2/3^‡^ | (67) | | 2/2 | (100) | 2/3^‡^ | (67) | | 0/2 | (0) | 1/3^‡^ | (33) | | 1/2 | (50) | 2/3^‡^ | (67) |
| Others (N=5) | 4/5 | (80) |  |  | | 5/5 | (100) |  |  | | 1/5 | (20) |  |  | | 4/5 | (80) |  |  |

AE, Adverse event.

^†^Classified according to CTCAE version 5

^‡^The number of cases including those with concomitant bowl obstruction (The patient records are duplicated.)
